# The complete chloroplast genome sequence of *Melampyrum koreanum* (Orobanchaceae), an endemic and hemi-parasitic herb in Korea

**DOI:** 10.1080/23802359.2021.1984330

**Published:** 2021-10-05

**Authors:** Dong-Pil Jin, Jin-Seok Kim, Yeon-Bong Ku, Chae Eun Lim

**Affiliations:** a Plant Resources Division, National Institute of Biological Resources, Incheon, South Korea; b Geumsugangsan, Uijeongbu, Korea

**Keywords:** Chloroplast genome, hemi-parasite, *Melampyrum*, Orobanchaceae, phylogenetic analysis

## Abstract

*Melampyrum koreanum* K.-J. Kim and S.-M. Yun 2012 (Orobanchaceae) is a hemi-parasitic herb, endemic to Korea. Here, the chloroplast genome of this species is reported. It was found to be 143,865 bp long, with a large single-copy region of 83,133 bp, a small single-copy region of 10,308 bp, and a pair of inverted repeat regions of 25,212 bp each. The chloroplast genome harbors 124 genes, including 79 protein-coding genes, 37 transfer RNA genes, and eight ribosomal RNA genes. Among the identified genes, *rpo*A and several *ndh* genes were determined to be pseudogenized due to the stop codon in the middle of the gene. The phylogenetic tree of the family was reconstructed based on 20 protein-coding genes, conserved across studied taxa. As a result, *Melampyrum* L. 1753 species were found to form a monophyletic group in the family.

Genus *Melampyrum* L. 1753 of family Orobanchaceae (formerly included in family Scophulariaceae) is distributed in Eurasia and North America, and members of this genus are characterized by a hemi-parasitic lifestyle (Olmstead et al. 2001; Choi 2018). To date, four *Melampyrum* taxa have been recognized in Korea, but various intermediate forms among species or lower taxonomic ranks have been observed (Choi 2018). Such morphological features obscure taxonomic delimitation.

In Korea, an endemic species, *Melampyrum koreanum* K.-J. Kim and S.-M. Yun 2012, was recorded based on a longer corolla tube and style than *Melampyrum roseum* Maxim. 1859 (Kim and Yun 2012). This species grows in the islands located in the southern sea of Korea. However, it is necessary to further study this species, because of known morphological variation within/among species (Choi 2018). In such cases, chloroplast (cp) genome data have been utilized as an evidence for inferring the phylogenetic relationship among taxa. Here we report, for the first time, the sequence of the cp genome of *M*. *koreanum*, and compare it with the previously reported cp genome of *M*. *roseum* (Li et al., 2019). In addition, we analyze the phylogenetic placement of *M*. *koreanum* within Orobanchaceae.

*M. koreanum* was collected from Hongdo island (Hongdo-ri, Heuksan-myeon, Sinan-gun, Jeollanam-do, Republic of Korea; 34°41′3″N, 125°11′53″E). The specimen (voucher number: NIBRVP0000806190) and DNA (NIBRGR0000806190) were deposited at the National Institute of Biological Resources (KB) (https://species.nibr.go.kr/index.do; Chang Woo Hyun (specimen), john0920@korea.kr; Jinwhoa Yum (DNA), lestes93@korea.kr). Genomic DNA was extracted from silica-dried leaves using a DNeasy Kit (QIAGEN, Seoul, South Korea) and paired-end (PE) libraries were prepared using a TruSeq nano DNA Kit (Illumina, San Diego, CA, USA) with a 550 bp average insert size. PE libraries were sequenced on a NovaSeq 6000 platform (Illumina). Approximately 289,653,358 of high-quality PE reads (2 × 150 bp) were generated and the reads were then mapped to the cp genome of *M. roseum* (GenBank: MN075942) using GetOrganelle (Jin et al. 2020). Genes in the cp genome of *M*. *koreanum* were annotated using GeSeq, with the *M*. *roseum* cp genome as a reference (Tillich et al. 2017). They were then manually edited by comparing with the genes of other genera, such as *Brandisia swinglei* Merr. 1918, *Euphrasia regelii* Wettst. 1896, *Lathraea squamaria* L. 1753, and *Neobartsia inaequalis* (Benth.) Uribe-Convers and Tank 2016, which are phylogenetically related (Bennett and Mathews 2006). Transfer RNAs (tRNAs) were confirmed using tRNAscan-SE (Lowe and Chan 2016).

Results showed that the cp genome of *M*. *koreanum* (MW463054) was 143,865 bp in length and quadripartite (large single-copy, 83,133 bp; small single-copy, 10,308 bp; a pair of inverted repeats, 25,212 bp each), with an overall GC content of 38.2%. The total number of genes in the cp genome was 124 (including 79 protein-coding genes, 37 tRNAs, and eight rRNAs), and coding regions covered 45.3% of the genome. When comparing with *M*. *roseum*, more tRNAs and pseudogenes were annotated, and duplicated *ycf*1 (partial gene) was excluded in *M*. *koreanum*. Six tRNA genes (*trn*D-GUC, *trn*fM-CAU, *trn*G-UCC, *trn*M-CAU, *trn*S-GCU, and *trn*S-GGA) and two pseudogenes were identified (*ndh*C and *ndh*D). In addition, some genes (*rpo*A, *ndh*B [×2], *ndh*J, and *ndh*K) annotated in *M*. *roseum* were determined as pseudogene due to stop codon in the middle of the gene. The sequences of the pseudogenes in *M*. *koreanum* were almost completely conserved in *M*. *roseum*. We found that many *ndh* genes, such as *ndh*A, *ndh*F, *ndh*G, *ndh*H, and *ndh*I, were missing from *M*. *koreanum.* Such extensive gene loss and/or pseudogenization of photosynthetic and photorespiratory genes have previously been reported in parasitic species (Cho et al. 2018; Zhou et al. 2019).

A maximum-likelihood (ML) tree of Orobanchaceae was constructed, based on 20 conserved coding regions to avoid biased topology by drastic variation among species, using IQ-TREE 1.6.12 (Trifinopoulos et al. 2016) with 1000 replicates (Figure 1). In this analysis, 31 species from Orobanchaceae and three species from a related family were included. The TVM + I + G model was selected as the substitution model, based on the Akaike information criterion, using jModelTest 2.1.6 (Darriba et al. 2012). On the ML tree, all nodes were supported with strong bootstrap values (>50%), and the members of Orobanchaceae are revealed as monophyletic. In this family, it seems that members have diverged from non-parasitic to holo-parasitic life cycles, which agrees with previous research findings (Samigullin et al. 2016; Cho et al. 2018; Zhou et al. 2019). However, a holo-parasitic plant, *L. squamaria*, is nested in a clade composed of hemi-parasitic plants (including *M. koreanum*), implying that the transition to obligate parasitism occurred in various lineages, rather than a single-stage (Bennett and Mathews 2006). *M. koreanum* is closely related to congeneric species, *M*. *roseum*. In future studies, the inclusion of additional cp genomes of other species would enable further elucidation of the phylogenetic relationships among Korean *Melampyrum* taxa.

**Figure 1.**
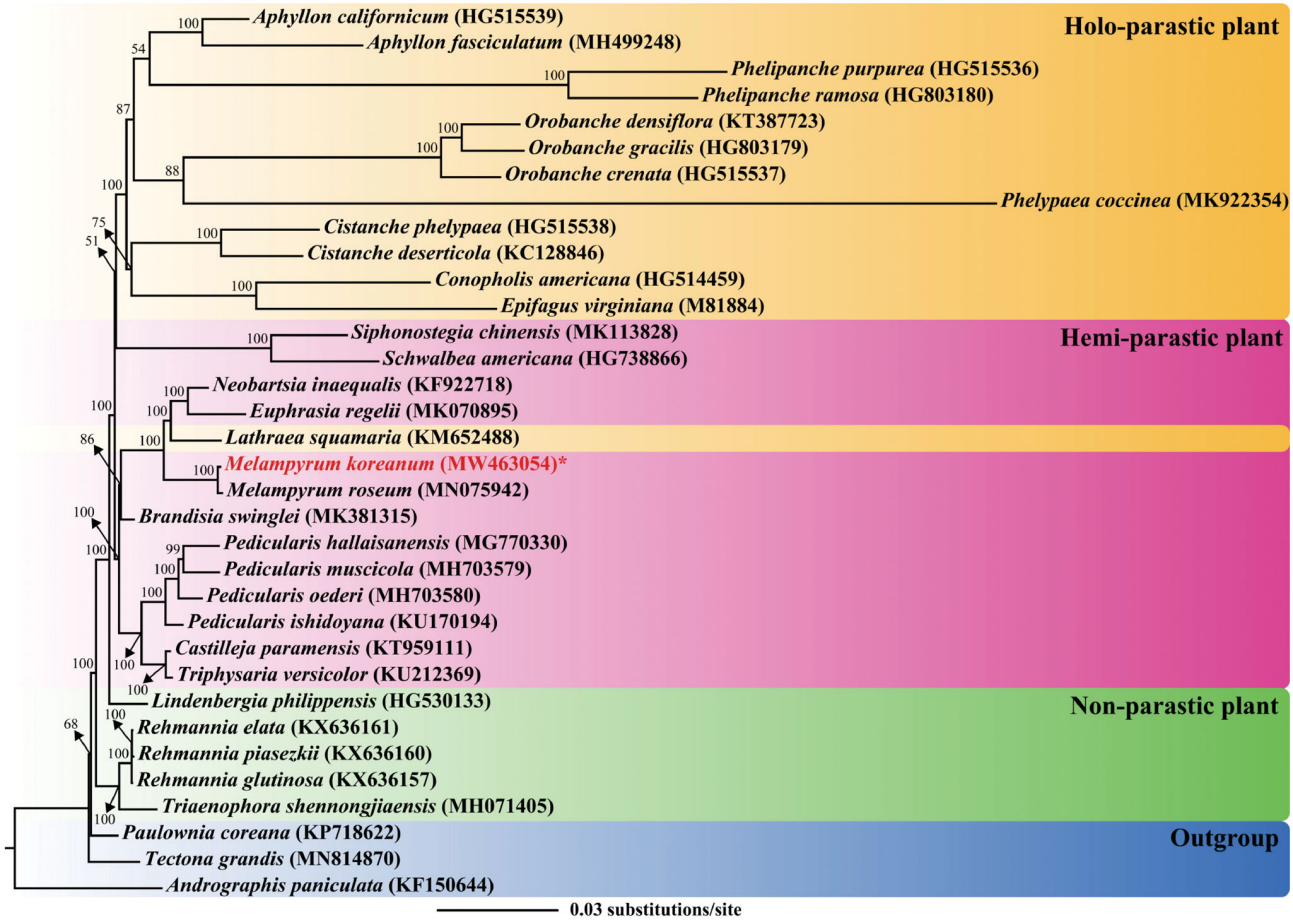
A maximum likelihood (ML) tree of Orobanchaceae, based on 20 coding genes of chloroplast genomes. The parasitic type of a species is represented by color rectangle: yellow, holo-parasitic plant; pink, hemiparasitic plant; green, non-parasitic plant. The number after the scientific name represents the GenBank accession of the species. The bootstrap value is shown on the node. The scale bar on the bottom indicates substitution per site. The newly sequenced individual is marked in red.

## Data Availability

The genome sequence data that support the findings of this study are openly available in GenBank of NCBI at https://www.ncbi.nlm.nih.gov/ under the accession no. MW463054. The associated BioProject, SRA, and Bio-Sample numbers are PRJNA733910, SRR14713231, and SAMN19462170, respectively.
